# Dioxaphosphorinane-Constrained Nucleic Acid Dinucleotides as Tools for Structural Tuning of Nucleic Acids

**DOI:** 10.1155/2012/215876

**Published:** 2012-10-24

**Authors:** Dan-Andrei Catana, Brice-Loïc Renard, Marie Maturano, Corinne Payrastre, Nathalie Tarrat, Jean-Marc Escudier

**Affiliations:** ^1^Laboratoire de Synthèse et Physicochimie de Molécules d'Intérêt Biologique, CNRS UMR 5068, Université Paul Sabatier, 31062 Toulouse, France; ^2^Université de Toulouse, INSA, UPS, INP, LISBP, 135 Avenue de Rangueil, 31077 Toulouse, France; ^3^INRA, UMR792 Ingénierie des Systèmes Biologiques et des Procédés, 31400 Toulouse, France

## Abstract

We describe a rational approach devoted to modulate the sugar-phosphate backbone geometry of nucleic acids. Constraints were generated by connecting one oxygen of the phosphate group to a carbon of the sugar moiety. The so-called dioxaphosphorinane rings were introduced at key positions along the sugar-phosphate backbone allowing the control of the six-torsion angles *α* to **ζ** defining the polymer structure. The syntheses of all the members of the D-CNA family are described, and we emphasize the effect on secondary structure stabilization of a couple of diastereoisomers of *α*,*β*-D-CNA exhibiting wether B-type canonical values or not.

## 1. Introduction

**Figure 1:**
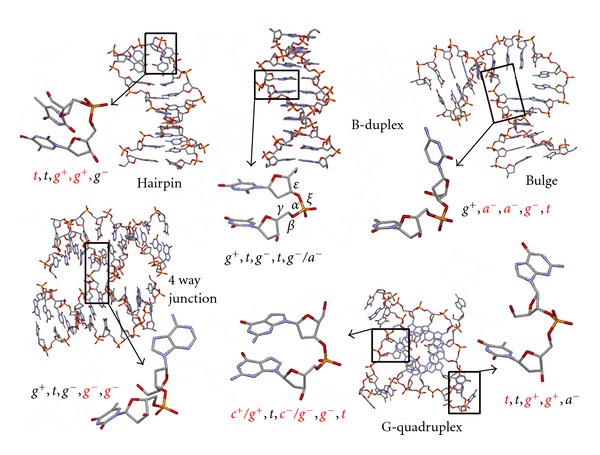
Examples of DNA secondary structures and associated backbone-torsion angles *γ*/*β*/*α*/*ε*/*ζ* of representative dinucleotide units. The following 6-fold staggered pattern of the torsional angles is used: *cis* = 0 ± 30° (c), *gauche*(+) = 60 ± 30° (g^+^), *anticlinal*(+) = 120 ± 30° (a^+^), *trans* = 180 ± 30° (*t*), *anticlinal*(−) = 240 ± 30° (a^−^), and *gauche*(−) = 300 ± 30° (g^−^). The notation g^−^/a^−^ is used to designate a torsion angle on the border of *gauche*(−) and *anticlinal*(−). PDB ID: Hairpin, 1ii1; duplex, 436d; Bulge, 1jrv; 4-Way junction, 1zez, and G-quad, 1kf1.

It is now clear that nucleic acids play several different roles in the living cell from genetic code storage to the catalysis of chemical reactions in ribosome. All of these particular behaviours are associated with various and very often transient structures of these polymers. The most prevalent secondary structure of nucleic acids is the double helix that can adopt either A- or B-type depending on the hydration level and/or the 2′-deoxyribosyl or ribosyl nature of the hybridized strands. While the backbone organization of double-stranded DNA and RNA is normally quite regular, there are many other secondary and tertiary structures that DNA and RNA molecules can adopt *in vivo *[1]. It is also well established that these disparate structures, which are predisposed to promote a significant local conformational heterogeneity in the sugar-phosphate backbone, play a crucial role in the fundamental biological processes where protein-nucleic acid interactions, folding, or catalytic activity are involved [2]. As a consequence nucleic acids can fold into biologically relevant distinct structures such as bulges, hairpin loops, *U*-turns, adenosine platforms, branched junctions, or quadruplexes (Figure 1). As proposed by few studies, the sugar/phosphate backbone of these unusual motifs exhibit a variety of conformations, which markedly differ from the regular conformational states of duplex DNA and RNA molecules [3–8]. However, the intrinsic role imparted to the phosphate diester backbone in respect with bases sequence in stamping these structures is still not properly defined. 

The determination of the precise biological role played by nonstandard helical conformations during the biochemically important processes (e.g., protein-DNA complexation, DNA processing, and DNA packaging) is also an area of intense study [9, 10]. An important study based on an analysis of available high-resolution crystallographic data and molecular simulation techniques has shown that, in contrast to free B-DNA structures, protein-bound B-DNA oligomers regularly involve noncanonical backbone geometries [11]. 

These unusual backbone states are believed to contribute to the specific recognition of DNA by proteins in assisting, at some stages, the fine structural adjustments that are required between DNA and proteins to form stable complexes. There are many examples in which DNA/protein complex formation results in DNA bending without disruption of the Watson-Crick base pairing [12–15]. Whereas this bending can be essential for complexes formation, it is generally sequence specific but with a strong impact on the sugar/phosphate backbone and can reach up to 90°. Unfortunately, experimental studies which aimed at determining the structural and functional implications of such helical deformations are somewhat complicated by the intrinsically transient nature of the corresponding backbone states. Stable structural analogues of these distorted backbone geometries would be very useful in the elucidation of the role that helical deformations play in nucleic acid interactions with proteins.

Mainly driven by the need of antisens research, most of the conformationally restricted oligonucleotides have been designed to enhance duplex formation ability and stability. Therefore, many efforts have been devoted to the synthesis of analogues with sugar-puckering conformational restriction of the North type [16–18, 18, 19]. To our knowledge, less attention has been paid to the design of conformationally restricted nucleosides with the aim of mimicking nucleic acid secondary structures containing non-Watson-Crick pairs or unpaired nucleotides. We are interested in the development of conformationally constrained dinucleotide building units in which the backbone torsional angles **α**–**ζ** can have predefined values that differ significantly from the typical values observed in DNA and RNA duplexes. In that context, the present paper will describe the last proposals and recent advances towards the introduction of conformational constraints into nucleotides by means of cyclic-phosphate structures. 

**Figure 2:**
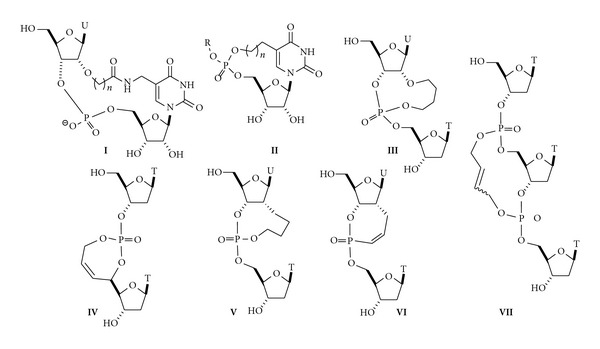
Selected macrocyclic-constrained nucleotides.

The introduction of constraint on the sugar-phosphate backbone by connecting a phosphate to a base, sugar moiety, or another phosphate of the same strand gave new opportunity to provide conformationally constrained nucleic acids mimics. The pioneering work of the Sekine's group in the late 90′s illustrated this approach. They were interested in developing mimics of the *U*-turn structure [20]. This sharply bent conformation has been commonly found in the anticodon loop of tRNAs and later discovered at the active site of hammerhead ribozymes. Therefore, they focused on the preparation of two cyclic diuridylates (compounds **I** and **II**, Figure 2), in which the two nucleosides moieties were connected either by an amide group or by a carbamate function for **I**, or by introducing a bridge between the 5′-phosphate group and the 5-*C* position of the uracil moiety for **II **[21]. When incorporated within oligonucleotides, these modified nucleotides **I** were both able to induce a severe bent into the oligomer, whereas the cyclouridilic derivatives of type **II** could either allow the formation of the duplex with the *R*_*P*_ configured phosphotriester moiety or be a good motif to mimic the *U*-turn structure with the *S*_*P*_ configured rigid-cyclouridilic acid derivative [22, 23]. 

Later on, the Poul Nielsen's group showed that the ring-closing metathesis (RCM) reaction was a suitable methodology towards the synthesis of conformationally restricted dinucleotide structures (compounds **III** to **VII**) in order to preorganize a single-stranded nucleic acid and to either form stabilized duplexes or to induce stabilization in other secondary structures [24–29]. The approach is based on the synthesis of dinucleotide units (or trinucleotide units) with a phosphotriester linkage constructed by RCM between an allyl-protected phosphate and another double-bond introduced at the appropriate location on the nucleoside either on the sugar or on the base moiety. Whereas all the constrained dinucleotide structures evaluated in duplex context showed destabilizing behavior, the *R*_*P*_ isomer of **V** provided the first example of stabilized three-way junction, in particular when the hairpin moiety was composed of ribonucleotides with an increase stability of +2.2°C rising to 2.7°C with the addition of Mg^2+^.

**Figure 3:**
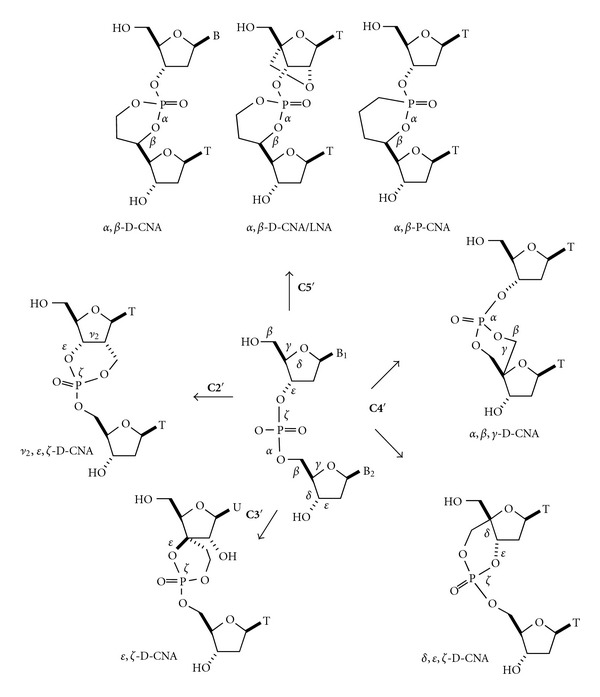
D-CNA dinucleotides building blocks for sugar/phosphate torsion angles control.

 The cyclic structures proposed there to modulate the sugar/phosphate backbone were composed of the smallest of a seven-membered cyclic phosphotriester to a very large macrocycle (up to eighteen members) and therefore exhibited rather flexible and undefined structures. In order to have a more rationalized approach to the design of covalently constrained nucleic acids (CNA) with specific canonical or noncanonical backbone conformations, we have developed dimeric building units in which two or three backbone torsion angles *α*−*ζ* are part of a well-defined six-membered ring structure (Figure 3).

The so-called D-CNAs are dinucleotides, in which a set of backbone torsion angles **α**–**ζ** is stereocontrolled to canonical or noncanonical values by a 1,3,2-dioxaphosphorinane ring structure. For a given dinucleotide step, there are fourteen possible [*β*-*D*-*deoxyribo*]-configured D-CNA stereoisomers which formally result from the introduction of a methylene or ethylene linker between a nonbridging phosphate oxygen and the 2′/4′-carbons (methylene linker) or the 3′/5′-carbons (ethylene linker) of the sugar moiety.

 Herein, we disclose the synthesis of each member of the D-CNA family, discuss their structural parameters which were established by means of X-ray diffraction analysis or NMR, and finally emphasize on the behaviour of *α*,*β*-D-CNA within duplex or hairpin secondary structure.

## 2. Synthesis of **α**,**β**-Constrained Nucleic Acids Dinucleotides (**α**,**β**-D-CNA and **α**,**β**-P-CNA)

**Scheme 1:**
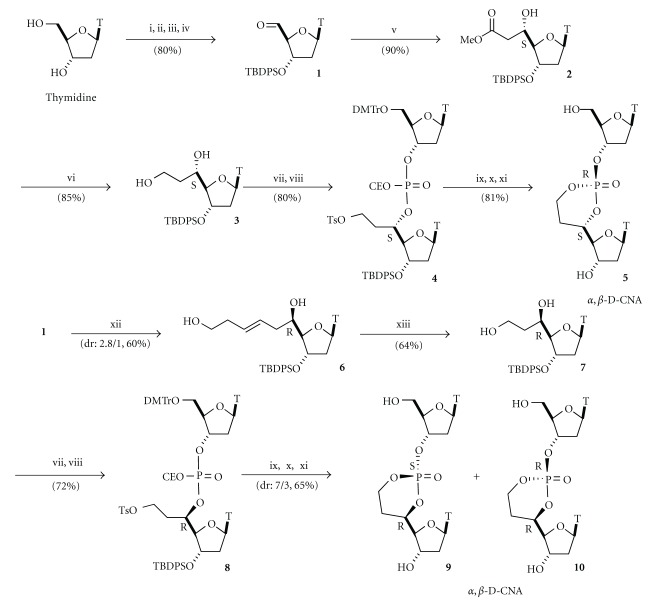
Synthesis of *α*,*β*-Dioxaphosphorinane-Constrained Nucleic Acid dinucleotides (*α*,*β*-D-CNA). *Reagents and conditions:* (i) TBDMSiCl, pyr, 95%; (ii) TBDPSiCl, imid, DMF, 97%; (iii) PTSA, MeOH, 95%; (iv) DCC, Cl_2_HCCOOH, DMSO then oxalic acid, 90%; (v) cat BiCl_3_, ZnI_2_, methyl acetate silylketene, DCM, 90%; (vi) NaBH_4_, EtOH, 85%; (vii) TsCl, CHCl_3_, pyr, 90; (viii) 5′-*O*-DMT-3′-*O*-diisopropylamino-cyanoethoxyphosphite thymidine, 1*H*-tetrazole, acetonitrile, then I_2_/H_2_O, 89%; (ix) Et_3_N, DMF, 90°C, 95%; (x) 3% TFA/DCM, 95%; (xi) TBAF, THF, 90%; (xii) (a) *ω*-*t*-butyldimethyl-silyloxy-allyltrimethylsilane, Et_2_O:BF_3_, DCM, (b) TiCl_4_, DCM, 60%; (xiii) (a) OsO_4_ cat, *N*-methylmorpholine *N*-oxide, H_2_O, (b) NaIO_4_, MeOH, (c) NaBH_4_, EtOH. 64%.

Our retrosynthetic analysis for the synthesis of *α*,*β*-D-CNA dinucleotides is based on the very simple strategy that consists of using both steric and anomeric effects to stereocontrol the cyclization reaction of a dinucleotide precursor, in which the phosphate oxyanions can attack an activated carbon atom. The preparations of the *α*,*β*-D-CNA dithymidine diastereoisomers are disclosed in Scheme 1 [30, 31]. 

 The key compounds of these pathways are the diastereopure 5′(*S*) and 5′(*R*)-*C*-hydroxyethyl-substituted nucleosides **3** and **7**, respectively. The former was obtained after reduction of the ester moiety of the product **2** of a diastereoselective Mukayama's reaction catalysed by BiCl_3_/ZnI_2_ on the aldehyde **1 **[32–34]. The starting aldehyde **1** was prepared by a Pfitzner-Moffatt oxidation procedure of the primary hydroxyl function of the thymidine after a classical three-step protection/deprotection sequence [35, 36]. The 5′(*R*) isomer **7** was generated from **1** through a Sakuraï's allylation with a *ω*-subsituted-allyltrimethylsilane [37, 38] followed by a three-step oxidative cleavage protocol of the double bond of the 5′-*C*-hydroxypentenyl-thymidine **6** isolated by silica gel chromatography from its diastereoisomer. Selective tosylation of the primary-hydroxyl function was achieved in good yield by reaction with tosyl chloride in the presence of pyridine [39] to provide the corresponding 5′-*C*-tosyloxyethylthymidines that were coupled with the commercially available thymidine phosphoramidite under a standard phosphoramidite procedure [40] to give the acyclic dinucleotides **4** and **8** after the oxidation step, respectively.

The cyclization reaction for the formation of the dioxaphosphorinane structure occurred by the treatment of **4** or **8** in basic medium to generate the phosphate anion that can displace the tosylate group. Surprisingly, the (*S*_*C*_, *R*_*P*_) isomer **5** of *α*,*β*-D-CNA was exclusively obtained from **4** whereas a lower stereoselectivity of 7/3 was observed for the formation of the (*R*_*C*_, *S*_*P*_) **9** and (*R*_*C*_, *R*_*P*_) **10** *α*,*β*-D-CNA from the 5′(*R*)-C precursor **8**. After deprotection of the hydroxyl functions both, *α*,*β*-D-CNA were structurally characterized and revealed that the major isomers were those with the dioxaphosphorinane ring in the chair conformation (See Section 6).

**Figure 4:**
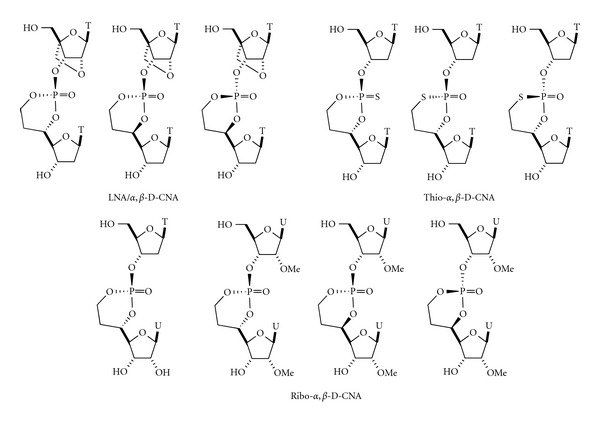
LNA-, thio-, and *ribo*-*α*,*β*-D-CAN.

Following the same chemical synthesis pathway, *α*,*β*-D-CNA analogues have been prepared by introducing a LNA-modified nucleoside during the phosphoramidite coupling to lead to LNA/*α*,*β*-D-CNA [41] (Figure 4), while changing the oxidation procedure from water/iodine to sulfur provided after cyclisation Thio-dioxa- and oxo-oxathiaphosphorinane structures (thio-*α*,*β*-D-CNA) [42]. Finally, starting from uridine or 2′-OMe-uridine, *ribo*-*α*,*β*-D-CNA could be achieved with the same diastereoselectivity outcome during the cyclisation process [43]. 

**Scheme 2:**
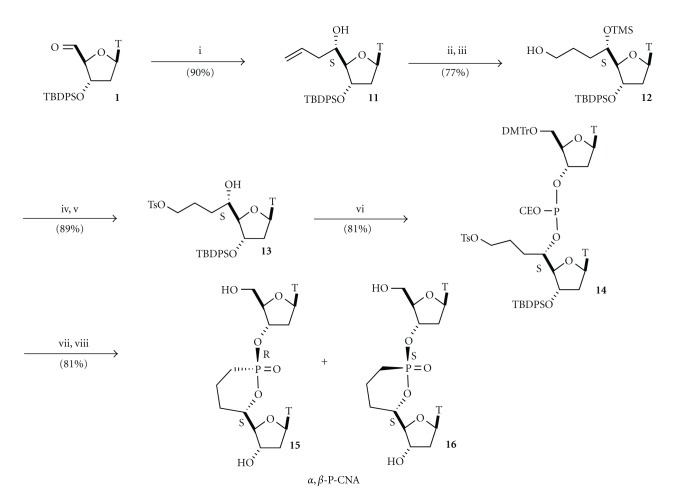
Synthesis of *α*,*β*-Phostone-Constrained Nucleic Acid dinucleotides (*α*,*β*-P-CNA). *Reagents and conditions:* (i) allyltrimethylsilane, BF_3_ : Et_2_O, 90%, (ii) Me_3_SiCl, pyr, rt, 3 h, 95%, (iii) BH_3_ : Me_2_S, THF, rt, 2 h then NaOH/H_2_O_2_, rt, 0.5 h, 90%, (iv) TsCl, CHCl_3_, pyr, rt, 16 h, 85%, (v) PTSA, MeOH, rt, 1 h, 90%, (vi) thymidine phosphoramidite,1H-tetrazole, rt, 81%, (vii) LiBr, acetonitrile, 90°C, MW 3 h, 85%, and (viii) 3%TFA, CH_2_Cl_2_, then TBAF, THF, rt, 1 h, 90%.

Therefore, the high diastereoselectivity observed for the formation of **5** led us to develop a strategy to synthesize the missing isomer in order to complete the set of CNA structural element. We turned our interest to phosphonate analogues of the D-CNA,, in which the dioxaphosphorinane ring would be replaced by a cyclic phosphonate called phostone providing Phostone-Constrained Nucleic Acids building blocks (P-CNA) [44]. We speculated that an intramolecular Arbuzov reaction, performed on the phosphite dinucleotide intermediate **14** similar to that prepared for the synthesis of D-CAN, would be suitable to reach this target (Scheme 2).

Starting from the thymidine aldehyde **1**, allylation under Hosomi and Sakurai's condition [45] gave pure 5′(*S*)-*C*-allylthymidine **11** that underwent a selective hydroboration/oxidation of the double-bond after protection of the secondary hydroxyl function to provide **12**. The required 5′-*C*-tosyloxypropylthymidine **13** was reached by tosylation of the primary hydroxyl function and removal of the trimethylsilyl protective group. The key phosphite intermediate **14** resulted from the standard coupling of **13** with the commercially cyanoethyl protected thymidine phosphoramidite using usual tetrazole activation and without oxidation step. In optimized Arbuzov reaction conditions (micro-waves irradiation and addition of LiBr), the cyanoethyl group was eliminated after the attack of the phosphorus on the activated carbon, leading to the formation of a 2/1 diastereoisomeric mixture of phostones. The removal of the 5′ and 3′-hydroxyl function protective groups and silica gel chromatography led to the isolation of the P-CNA **15** and **16**. The (*S*_*C*_, *S*_*P*_) *α*,*β*-P-CNA isomer **16** was isolated as the minor isomer and was corresponding to the structural analogue of the missing *α*,*β*-D-CNA.

## 3. Synthesis of **α**,**β**,**γ**-Constrained Nucleic Acids Dinucleotides (**α**,**β**,**γ**-D-CNA) and **δ**,**ε**,**ζ**-Constrained Nucleic Acids Dinucleotides (**δ**,**ε**,**ζ**-D-CNA)

**Scheme 3:**
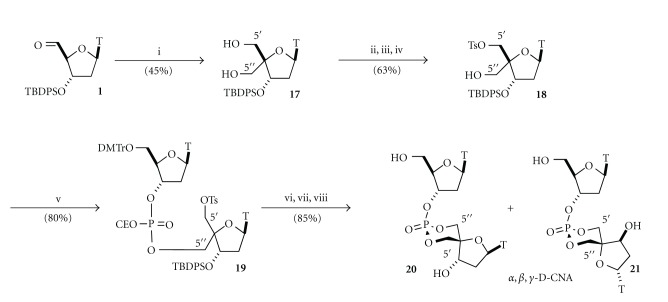
Synthesis of *α*,*β*,*γ*-Dioxaphosphorinane-Constrained Nucleic Acid dinucleotides (*α*,*β*,*γ*-D-CNA). *Reagents and conditions:* (i) HCOH, 2N NaOH, dioxane then NaBH_4_, 45%, (ii) DMTrCl, pyr, 70%, (iii) TsCl, CHCl_3_, Pyr, 90%, (iv) 3% TFA, DCM, quant. (v) 5′-*O*-DMT-3′-*O*-diisopropylamino-cyanoethoxyphosphite thymidine, 1*H*-tetrazole, acetonitrile, then I_2_/H_2_O, 80%, (vi) K_2_CO_3_, DMF, 90°C, 100%, (vii) TBAF, THF, 85%, and (viii) 3% TFA/DCM, quant.

**Scheme 4:**
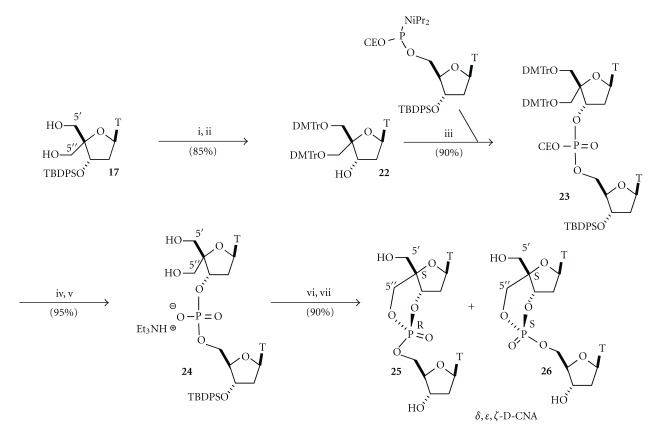
Synthesis of *δ*, *ε*, *ζ*-Dioxaphosphorinane-Constrained Nucleic Acid dinucleotides (*δ*,*ε*,*ζ*-D-CNA). *Reagents and conditions:* (i) DMTrCl, DMAP, pyr, 90%, (ii) TBAF, THF, 95% and (iii) 5′-thymidine phosphoramidites, 1*H*-tetrazole, acetonitrile, then I_2_/H_2_O, 90%, (iv) 3% TFA, DCM, 95%, (v) Et_3_N, acetonitrile, 60°C, quant, and (vi) 1-(mesitylene-2-sulfonyl)-3-nitro-1,2,4-triazole (MSNT), pyr, 80°C, quant, (vii) TBAF, THF, 90%.

These two representatives of the D-CNA family originate from the connection of the phosphate to the 4′-*C*-carbon atom either of the downstream sugar moiety for the *α*,*β*,*γ*-D-CNA or of the upstream sugar moiety in the case of the *δ*,*ε*,*ζ*-D-CNA (Figure 3) [46]. Therefore, their synthesis started from a common intermediate 4′-*C*-hydroxymethyl-thymidine **17** obtained by a treatment of the thymidine aldehyde **1** under Cannizzaro's conditions (Schemes 3 and 4) [47]. 

In the case of *α*,*β*,*γ*-D-CNA thymidine dinucleotides, the dioxaphosphorinane ring structure was formed as previously described for *α*,*β*-D-CNA by displacement of a tosyl group by a phosphate anion generated by the removal of a phosphate cyanoethyl protective group in basic medium (Scheme 3). The acyclic precursor involved is the dithymidine **19** prepared by coupling 5′-*O*-tosyl-4′-*C*-hydroxymethylthymidine **18 **with the commercially available thymidine phosphoramidite using standard phosphoramidite technology. A three-step procedure involving first a selective protection of the 5′′-hydroxyl function of **17**, followed by tosylation of the residual primary 5′-hydroxyl function, and finally removal under acidic conditions of the dimetoxytrityl group furnished the required 5′-*O*-tosyl-4′-*C*-hydroxymethyl thymidine **18**. The removal of the cyanoethyl group from **19** by treatment with potassium carbonate in dimethylformamide generated the phosphate anion which by heating at 90°C provided the formation of two *cis-* and *trans-* isomers of protected *α*,*β*,*γ*-D-CNA in a 2/1 ratio in favor of the *cis.* After the removal of the 5′- and 3′-protective groups, the *α*,*β*,*γ*-D-CNA *cis* **20** could be separated from the *trans* isomer **21** and characterized.

 A similar approach towards the synthesis of *δ*,*ε*,*ζ*-D-CNA, that is, introduction of a tosyl group on the 5′′-hydroxyl function, phosphoramidite coupling, and nucleophilic attack of the phosphate has been investigated, but it turned out to be troublesome and the desired 5′′-*O*-activated nucleoside could only be obtained in very poor yield. Therefore, we choose to use the well-known phosphotriester methodology that allows the formation of a phosphoester from a phosphate with an alcohol in the presence of an activator such as 1-(mesitylene-2-sulfonyl)-3-nitro-1,2,4-triazole (MSNT) [48]. 

Starting from the diol **17**, both primary hydroxyl functions were protected as dimethoxytrityl ether and the 3′-*O*-silyl protective group was removed by treatment with fluoride ion to produce the nucleoside **22** (Scheme 4). A phosphoramidite coupling with a 5′-*O*-phosphoramidite-thymidine gave dinucleotide **23** that was consecutively treated in acidic medium to remove the dimethoxytrityl protective groups and in basic medium with triethylamine to eliminate the cyanoethyl phosphate protective group. The key phosphodiester **24** was then available to undergo the cyclisation process according to the phosphotriester methodology. Even if two primary hydroxyl functions were present, only the 5′′-hydroxyl reacted under the MSNT catalyst to form the dioxaphosphorinane ring. This high regioselectivity was unfortunately combined with no diastereoselectivity in the neither ring formation nor the formation of a 1/1 mixture of (*S*_*C*4′_, *R*_*P*_) and (*S*_*C*4′_, *S*_*P*_) diastereoisomers **25** and **26**, respectively. The poor diastereoselectivity could be explained by the fact that due to the fused sugar ring none of these compounds feature a chair conformation of the dioxaphosphorinane structure, which is indicative that there is not a more favorable intermediate during the cyclisation process.

## 4. Synthesis of ***ν***_2_,**ε**,**ζ**-Constrained Nucleic Acids Dinucleotides (***ν***_2_,**ε**,**ζ**-D-CNA)

**Scheme 5:**
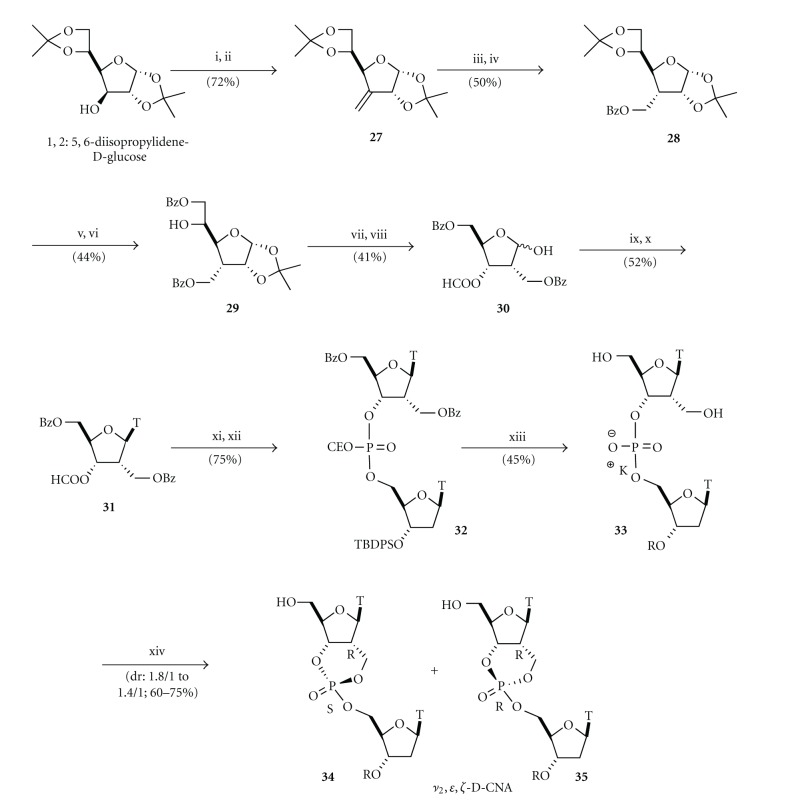
Synthesis of *ν*_2_,*ε*,*ζ*-Dioxaphosphorinane-Constrained Nucleic Acid dinucleotides (*ν*_2_,*ε*,*ζ*-D-CNA). *Reagents and conditions*: (i) PDC, Ac_2_O, DCM, 80°C, 87%; (ii) nBuLi, Ph_3_PMeBr, THF, 83%; (iii) BH_3_Me_2_S, THF, 0°C then H_2_O_2_ 25%, NaOH 2 N, H_2_O/THF (1/1), 81%; (iv) BzCl, pyr, DMAP, rt, 61%; (v) HCl 0.06 N, MeOH, 55°C, 2 h, 81%; (vi) BzCl, pyr, 0°C, 2 h, 54%; (vii) AcOH 80%, 80°C, 46%; (viii) KIO_4_, EtOH/H_2_O (1/1), rt, 18 h, 90%; (ix) Ac_2_O, pyr, 94%; (x) TMSOTf 0.55 M in toluene, bis(trimethylsilyl)thymine, acetonitrile reflux, 56%; (xi) NH_4_OH 28%, rt, quant; (xii) 3′-*O*-*t*-butyldiphenylsilylthymidine-5′-*O*-phosphoramidite, 1*H*-tetrazole, acetonitrile, then collidine, I_2_/H_2_O, 75%; (xiii) K_2_CO_3_, MeOH/H_2_O (4/1), rt, 6 h, 45%; (xiv) 1-(mesitylene-2-sulfonyl)-3-nitro-1,2,4-triazole (MSNT), pyr, rt, 60–75%.

The synthesis of *ν*_2_,*ε*,*ζ*-D-CNA implied the connection of the phosphate to the 2′-*C*-carbon of the sugar moiety through a methylene link [49]. To achieve this goal, instead of starting from a nucleoside precursor, we choose to reproduce a protocol previously described by Marquez and Coll. that used the commercially available 1,2 : 5,6-diisopropylidene-*D*-*glucose* and through an elegant rearrangement gave the pivotal protected 2-deoxy-*C*-(hydroxymethyl)-*D*-*ribo*furanose **30** (Scheme 5) [50]. Then a Vorbrüggen et al.'s procedure [51] could install the thymine base and a phosphoramidite coupling would provide the dinucleotide that could undergo the dioxaphosphorinane ring formation, here again by the phosphotriester method leading to the target *ν*_2_,*ε*,*ζ*-D-CNA. 

The secondary 3-hydroxyl function of 1,2 : 5,6-diisopropylidene-*D*-*glucose* was oxidised to ketone to be substrate for a Wittig homologation with methyltriphenylphosphonium on the 3-*C* position providing the sugar **27** with an exocyclic double bond. The hydroxymethyl function at 3-*C* was generated by a hydroboration/oxidation that occurred from the top-face of the sugar resulting in the formation of the required *R*-configured 3-carbon. Benzoylation of the resulting hydroxyl function provided the fully protected 3-deoxy-3-hydroxymethyl-*D*-*allose* **28**. Acidic hydrolysis of the 5,6-isopropylidene followed by a tricky selective benzoylation of the primary hydroxyl function led to **29** with the unprotected 5-secondary alcohol. Acetolysis of the 1,2-isopropylidene gave the 6-*O*-benzoyl-3-deoxy-3-benzoyloxymethyl-*D*-*allose* that was subsequently treated with sodium periodate to cleave the diol system. After rearrangement, the 2-deoxy-2-benzoyloxymethyl-*D*-*ribose* analogue has been isolated as a mixture of anomers. After protection of the anomeric position with an acetate function, thymine was introduced by a Vorbrüggen's procedure and the thymidine analogue **31** was obtained in a 1/9 ratio of *α*/*β* anomers. Removal of the residual-formyl group by aqueous ammonia gave the suitable nucleoside for a phosphoramidite coupling with a 5′-*O*-phosphoramidite-thymidine ending in the formation of the acyclic dinucleotide **32**. Potassium carbonate treatment, to remove the base labile benzoyl and cyanoethyl protective groups proceeded with a concomitant loss of the *t*-butyldiphenylsilyl group and dinucleotide **33** was obtained as a 1/1 mixture of fully deprotected, and 3′-*O*-silylated dinucleotide. These dinucleotides were separated and submitted to the cyclisation activated by 1-(mesitylene-2-sulfonyl)-3-nitro-1,2,4-triazole (MSNT) to furnish (*R*_*C*2′_, *S*_*P*_) and (*R*_*C*2′_, *R*_*P*_) *ν*_2_,*ε*,*ζ*-D-CNA **34** and **35**, respectively. While the ring formation occurred with a 1/1.8 ratio in the case of the partially protected dinucleotide, the diastereoselectivity was lowered to 1/1.4 for the fully deprotected dinucleotide. 

## 5. Synthesis of *xylo*-**ε**,**ζ**-Constrained Nucleic Acids Dinucleotides (*xylo*-**ε**,**ζ**-D-CNA)

**Scheme 6:**
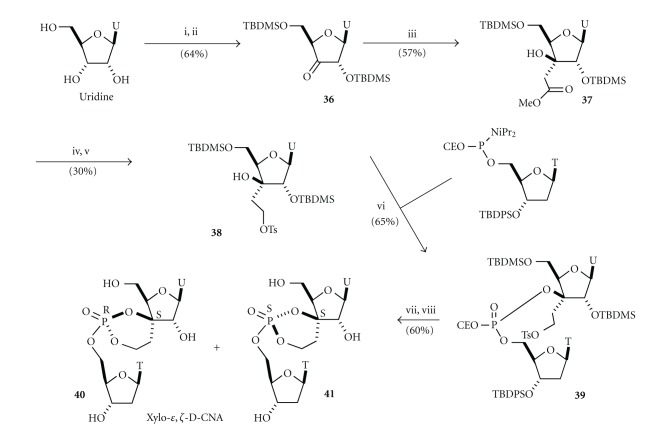
Synthesis of *xylo*-*ε*,*ζ*-Dioxaphosphorinane-Constrained Nucleic Acid dinucleotides (*xylo*-*ε*,*ζ*-D-CNA). *Reagents and conditions:* (i) TBDMSCl, AgNO_3_, Pyr, THF, 74%, (ii) Dess-Martin periodinane, Pyr, DCM, rt, 87%, (iii) *t*-butyldimethylsilyl-methyl-ketene, BF_3_·Et_2_O, DCM, −78°C, 57%, (iv) DIBAH, DCM, 0°C, 2 h then rt 12 h, 46%, (v) TsCl, Pyr, CHCl_3_, 64%, (vi) 3′-O-*t*-Butyldiphenylsilylthymidine-5′-*O*-phosphoramidite (2 eq), tetrazole, acetonitrile then collidine, I_2_/H_2_O, 65%, (vii) K_2_CO_3_, DMF, rt., 81%, and (viii) TBAF (3.3 eq), THF, rt, 74%.

The restrains on only the torsional angles *ε* and *ζ* requires the formation of a spiro connection between the sugar and the dioxaphosphorinane rings by introduction of an ethylene linker between the 3′-*C*-carbon atom of the sugar moiety and the phosphate (Figure 3) [52]. To date, on the four possible stereoisomers, we have reported the synthesis of the *xylo*-configured D-CNAs because they represent a class of distorted structures directly available from commercially uridine (Scheme 6). A similar approach to that proposed for *α*,*β*-D-CNA has been followed for the preparation of the *xylo*-*ε*,*ζ*-D-CAN, that is, aldol condensation to introduce the ethylene link on the 3′-*C* and activation through a tosylation to form the dioxaphosphorinane ring after phosphoramidite coupling.

Uridine was selectively protected on the 5′-*O* and 2′-*O* by *t*-butyldimethylsilyl group following the Ogilvie's procedure [53] before being oxidized the with Dess-Martin periodinane [54] to give the keto-uridine **36**. A stereoselective Mukaïyama's addition of the *t*-butyldimethylsilyl-methyl-ketene acetal occurred on the *Re* face of the carbonyl as determined by NOE experiments on the adduct **37**. Reduction of the ester function turned to be rather difficult using NaBH_4_ and the solution came from DIBAH; however; in a modest yield. The primary hydroxyl function was then selectively tosylated to provide the 3′-tosyloxyethyl-*xylo*-uridine **38** suitable to be engaged in the phosphoramidite coupling with the 5′-*O*-phosphoramidite-thymidine. The acyclic 3′-*C *-tosyloxyethyluridine/thymidine dinucleotide **39** was then submitted to basic treatment at room temperature, and the generated phosphate anion cleanly displaced the tosyl group to form a 1/1 diastereoisomeric mixture of protected (*S*_*C*3′_, *R*_*P*_) and (*S*_*C*3′_, *S*_*P*_) *xylo*-*ε*,*ζ*-D-CNA **40** and **41**, respectively, which have been separated on reverse phase HPLC after deprotection. Whereas a relative instability of the phosphotriester could be expected due to the presence of the secondary hydroxyl function, the spiro structure with an “*S*” configuration of 3′-*C* fixed their relative positions away to the necessary “on line” conformation avoiding any trans-esterification process [55, 56]. 

## 6. Structural Assignment

The determination of the values of the constrained torsional angles within D-CNA structures relied on the establishment of the geometry of the dioxaphosphorinane ring whether in chair conformation or not. Some of D-CNAs were crystallized and solid phase structures were determined by X-ray diffraction analysis for (*R*_*C*5′_, *S*_*P*_) *α*,*β*-D-CNA TT (compound **9**, Scheme 1), (*S*_*C*5′_, *R*_*P*_) *α*,*β*-D-CNA TU (Figure 4), and (*S*_*C*4′_, *S*_*P*_) *δ*,*ε*,*ζ*-D-CNA TT (compound **26**, Scheme 4). Moreover, NMR analysis of the H/H and H/P coupling constants of the protons involved in the dioxaphosphorinane ring or in the sugar moieties either corroborated the results of the X-ray analysis or allowed for the establishment of the rings conformations. Interestingly, ^3^ *J*_H/P_ coupling constants between relevant protons within the dioxaphosphorinane ring and the phosphorous gave important information because they exhibit specific values dependant on the relative axial or equatorial position of the proton within the six membered ring, that is, ^3^ *J*_Hax/*P*_ < ca · 3 Hz and ^3^ *J*_Heq/*P*_ < ca · 20 Hz, respectively [57]. Therefore, a careful examination of these data allowed for the determination of the dioxaphosphorinane ring conformation, whereas ^3^ *J*_H/H_ coupling constants gave also information on the sugar puckering. The conformational ranges of the constrained torsional angles within D-CNA determined by these methods are summarized in Table 1. Torsional angles' values depicted in A- or B-type duplex are given as reference and are considered as canonical values for the regular double-helix structure [58]. 

**Figure 5:**
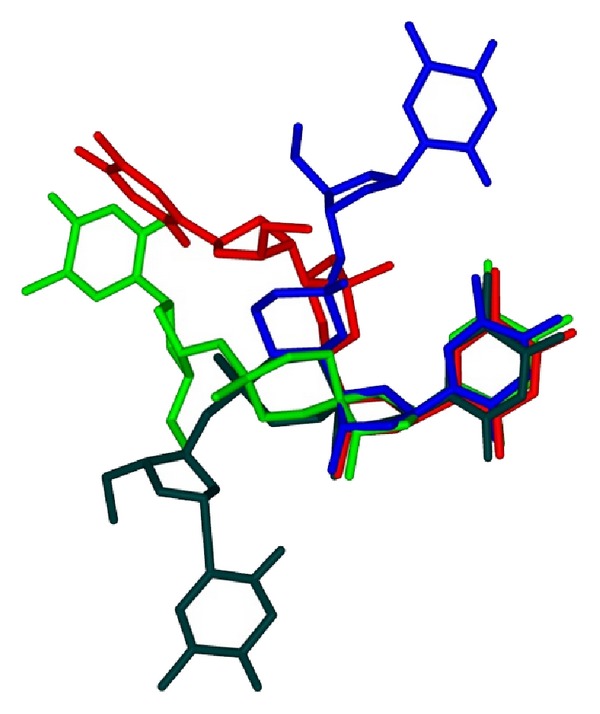
superimposition of minimized structures of *α*,*β*-D-CNA (*g* ^−^, *t*) blue; *α*,*β*-D-CNA (*g* ^+^, *t*) red; *α*,*β*,*γ*-D-CNA (*g* ^−^, *g* ^−^, *g* ^−^) green; *α*,*β*,*γ*-D-CNA (*g* ^+^, *c*/*g* ^+^, *g* ^−^/*a* ^−^) dark green.

 Among all the sets of constrained torsional angles, the values exhibited for *α* and *β* by the (*R*_*C*5′_, *S*_*P*_) isomer of *α*,*β*-D-CNA (or its analogue LNA/*α*,*β*-D-CNA) are identical to those observed for the A- or B- type duplex. In contrast, and as expected by the proposed approach, all the others constrained dinucleotides feature-torsional angle' values greatly differ from the canonical ones. Therefore, these members offer an extraordinary diversity in the relative spatial arrangement of the bases moieties allowing the description of an unusual local shape of nucleic acids. In order to illustrate this point, Figure 5 shows a superimposition of (*R*_*C*5′_, *S*_*P*_)-, (*S*_*C*5′_, *R*_*P*_) *α*,*β*-D-CNA and *cis*-, *trans*-*α*,*β*,*γ*-D-CNA featuring a (*g* ^−^, *t*), (*g* ^+^, *t*), (*g* ^−^, *g* ^−^), and (g^+^, c/g^+^) set of value for *α* and *β*, respectively. Whereas (*R*_*C*5′_, *S*_*P*_) *α*,*β*-D-CNA analogue stands for a good mimic of B-type dinucleotide with the thymine bases mostly stacked, it is nicely illustrated that the two bases can be oriented in rather different planes in the others D-CNA dinucleotides. 

The sugar puckering of each nucleosides within D-CNA was estimated by the empirical equation of Altona-Sundaralingan using the ^3^ *J*_H/H_ coupling constants: C2′-*endo* (%) = [*J*_1′/2_/(*J*_1′/2_′ + *J*_3′/4_′)] × 100 [59]. Due to an increase of the electronegativity of the 3′ oxygen by introduction of a neutral internucleotidic linkage, the sugar pucker of the upstream nucleoside is favored in its C2′-*endo* conformation (South) [60]. The determination of the impact that a neutral phosphotriester linkage would display in the conformational North/South equilibrium is particularly important as it is well recognized that this conformational state is of major importance for the DNA duplex formation ability.

The examination of the relevant coupling constants showed that in all cases for D-CNA built with 2′-deoxyribose, the sugar puckering of the 5′-upstream or 3′-downstream nucleoside were in the C2′-*endo* conformation. However, for *α*,*β*-, *α*,*β*,*γ*-, and *ν*_2_,*ε*,*ζ*-D-CNA the 5′-upstream nucleoside sugar puckering equilibrium was strongly displaced toward the C2′-*endo* conformation (South) compared to natural 2′-deoxyribose units [61]. Interestingly, in the cases of *ribo*-*α*,*β*-D-CNA (Figure 4), even the 2′-OMe-*ribose* unit was pushed into the C2′-endo conformation upon the influence of the neutralized internucleotidic linkage. Only the *xylose*-configured sugar within *xylo*-*ε*,*ζ*-D-CNA adopted a North conformation (C2′-*exo*).

Dioxaphosphorinane-modified sugar/phosphate backbone of dinucleotide could therefore represent a promising methodology to provide alternative backbone conformations. It is likely that D-CNA within DNA or RNA oligomers would be able to modulate the shape and the folding with significant-conformational distortion of secondary nucleic acid structures.

## 7. Survey of **α**,**β**-D-CNA Dinucleotides Behavior within Oligonucleotides

We focused our interest on the study of the impact of the restraint on one specific torsional angle, *α*, through the behavior within oligodeoxynucleotide (ODN) of a couple of *α*,*β*-D-CNA diastereoisomers featuring either canonical or noncanonical *α*/*β* combination [62]. As shown previously (Scheme 1 and Table 1), (*S*_*C*5′_, *R*_*P*_) and (*R*_*C*5′_, *S*_*P*_) *α*,*β*-D-CNA derivatives **5** and **9** can be easily prepared and their structural assignment showed that the (*R*_*C*5′_,  *S*_*P*_) *α*,*β*-D-CNA **9** exhibited a canonical value set (*gauche*(−), *trans*) for *α* and *β*, whereas its diastereoisomer (*S*_*C*5′_, *R*_*P*_) *α*,*β*-D-CNA **5** differed only on the *α* value which was changed to the *gauche*(+) conformation while maintaining *β* in the *trans* configuration. Therefore, we dispose of a unique couple of modified nucleotides that will give us new insight on the impact of backbone preorganization either in the B-type duplex geometry or with a strong torsional stress applied on *α* corresponding to that observed in DNA/protein complex or in unpaired secondary structures such as hairpin or bulges.

**Figure 6:**
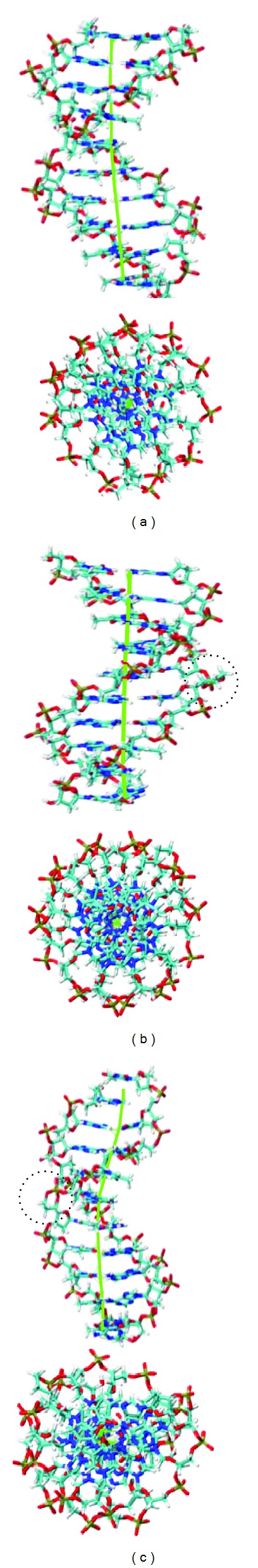
Side and top views of B-type duplex produced by molecular dynamics simulations, the double-helix axes are outlined in green. (a) Unmodified dA_10_/dT_10_ **ODNref**, (b) dA_10_/dT_4_ ***TxT***T_4_ **ODNgm** with ***TxT*** = (*R*_*C*5′_, *S*_*P*_) *α*,*β*-D-CNA TT, or (c) **ODNgp** with ***TxT*** = (*S*_*C*5′_, *R*_*P*_) *α*,*β*-D-CNA TT.

A molecular dynamic simulation has been run on dA_10_/dT_10_ duplex whether modified or not with one TT step constrained with (*R*_*C*5′_, *S*_*P*_) or (*S*_*C*5′_, *R*_*P*_) *α*,*β*-D-CNA denoted as **ODNref**, **ODNgm**, and **ODNgp**, respectively (Figure 6) [63]. This study gave us two main results: compared to unmodified duplex the structure seems to accommodate the canonical restraint on *α* with a straightness of the double-helix whereas the *gauche*(+) conformation induced a bend without loss of the Watson-Crick base pairing. Therefore, these observations let us speculate that controlling the torsion of an ODN into its B-type canonical form should enhance the duplex formation ability, whereas displacing it to around +70° might result in the formation of localized distortion able to stabilize unpaired conformations.

Interestingly, analysis of the atomic fluctuations derived from these simulations indicated that in **ODNgm** all of these fluctuations were diminished in both strands which could be indicative of a potential duplex stabilisation, whereas in **ODNgp** they were unchanged compared to those observed in **ODNref**.

Therefore, we investigated the behavior of *α*,*β*-D-CNA within ODNs by thermal denaturation studies by means of UV experiments. Selected results are reported in Table 2 for (*R*_*C*5′_, *S*_*P*_) *α*,*β*-D-CNA (denoted to as **CNAgm**), in Table 3 for a comparative study between (*R*_*C*5′_, *S*_*P*_) and (*S*_*C*5′_, *R*_*P*_) *α*,*β*-D-CAN, and in Table 4 for hairpin structures stabilisation by (*S*_*C*5′_, *R*_*P*_) *α*,*β*-D-CNA (denoted to as **CNAgp**). 

All the ODNs containing D-CNA were obtained by automated synthesis according to the phosphoramidite methodology. The phosphoramidite building blocks of (*R*_*C*5′_, *S*_*P*_) and (*S*_*C*5′_, *R*_*P*_) *α*,*β*-D-CNA were synthesized by conventional method and their incorporation within ODNs occurred similarly to standard phosphoramidite with no change in automated synthesis protocols but with a smooth deprotection in ammonia at room temperature.

 The introduction of a canonical constraint within ODN resulted in a remarkable stabilizing effect on duplex formed with DNA counterparts (Δ*Tm* = +5.0 ± 1°C/mod, Table 2, entries 2, 6–10) [64]. These increases in Tm values are insensitive to salt concentration suggesting that the effects observed were primarily conformational rather than electrostatic. Thus, **CNAgm** represents a rare example of constrained nucleotide that significantly increases the hybridizing properties of ODNs without forcing the sugar pucker into the C3′-*endo* conformation, demonstrating that the preorganization concept can also be successfully applied to other torsional angles than those involved in the sugar moiety puckering. The ability of **CNAgm** to adapt to the B-conformation of the double-helix is outlined by its additive stabilizing effect (entries 2–4) when included in the same strand and also when the two strands are modified with one **CNAgm**, with a maximum effect when constraints were close to the 3′-ends preventing end frying (entries 11–14).

It is noteworthy that a rather moderate effect was observed with RNA counterparts (Δ*Tm* = +0.9 to +3.0°C/mod), which could originate from the reluctance of the upstream-furanose unit of the *α*,*β*-D-CNA to undergo a significant conformational change from 2′-*endo* to 3′-*endo* in the hybrid duplex DNA/RNA due to the loss of the internucleotidic negative charge. 

 On the other hand, when we prepared sequences either modified by **CNAgm** or by **CNAgp **we were able to have insight on the cost in terms of thermal stability of a dramatic change of restraint on *α* from *gauche*(−) to* gauche*(+). As expected, incorporation of **CNAgp** featuring noncanonical (*gauche*(+), *trans*) *α*/*β* combination resulted in an important loss in duplex stability (−4.2° to −13.6°C/mod, Table 3) depending on the sequence length and composition. Short decamer and rather unstable oligothymidilate exhibited the higher destabilized level (entries 1–3) while increasing the size to 18-mer (entries 4–10) and 24-mer (entries 11, 12) modulated the impact of the *gauche*(+) restraint around −5 ± 1°C/mod and also minimized the positive effect on duplex formation ability of **CNAgm** from +5 ± 1°C/mod to +1 ± 1°C/mod. We showed that regardless of the type of restriction applied to ODN a high level of sequence discrimination was maintained as natural duplexes do.

Interestingly, exceptions in the destabilization effect of **CNAgp** (entries 6 and 13) and in the positive impact of **CNAgm **(entry 13) appeared. However, the first sequence is hemipalindromic and the second is fully self-complementary. Therefore, they can exist either as duplex or as hairpin structure. A further experiment showed that the observed transitions were indeed a combination of melting temperature from equilibrium made of high hairpin transition and lower duplex melting. In the case of the Drew-Dickerson sequence (entry 13), **CNAgm** was able to displace the equilibrium in favor of the duplex, whereas **CNAgp **displaced it to the hairpin structure because constraint was imposed within the loop.

In order to emphasize the effect of stabilization of unpaired region of secondary nucleic acids structure by **CNAgp**, we engaged the synthesis of modified *T*_4_ loop within hairpin that could differ in their stem composition and especially in the AT or CG loop-closing base pair (Table 4) [65]. 

In a hairpin, which is a single-stranded structure, the sugar/phosphate backbone orientation is reversed by means of the loop moiety. The necessary torsional stress is not spread throughout all the loop constituents but ensured by a sharp-turn position called the “turning phosphate” that displays a *gauche*(+) transition of *α* torsional angle [66]. Therefore, it was tempting to speculate that **CNAgp** could play the role of a preorganized “turning phosphate” and as a consequence could induce hairpin stabilization [67]. **CNAgp** was installed in all the possible positions within the loop, and the thermal stabilities were evaluated by UV melting curves analysis. In the case of *T*_4_-looped hairpin structures with AT closing base pair, the central position was best suited for **CNAgp** (Table 4, entry 3 versus 2, 4, and 5) with a maximum in Δ*T*m of +3.0°C. However, if a constraint in the middle of the loop helped the hairpin folding, when installed at the 3′-end of the loop, **CNAgp** strongly was destabilized by −7.0°C (entry 5). Remarkably, with CG closing base pair, **CNAgp** behaves as a stabilizing analogue in any position within the loop (entries 6–9). Indeed, circular dichroism experiments showed that when **CNAgp** was placed in the middle of the loop (entry 8), the stem structure was not altered, whereas when located in 3′-end region a stem rearrangement occurred that could participate to the stabilization enhancement observed (entry 9). Similar results were depicted for *T*_5_-looped hairpin structures with Δ*T*m up to +5.0°C [68]. 

Eventually, we showed that the two diastereoisomers of *α*,*β*-D-CNA featuring a fixed torsional angle alpha either in the B-type canonical value *gauche*(−) or in atypical *gauche*(+) conformation are powerful building blocks allowing high level of duplex or hairpin stabilization as expected according to the preorganization concept. This is evidence that controlling the sugar/phosphate backbone not only in terms of sugar puckering is a promising approach toward the control of nucleic acids secondary structures.

## 8. Conclusions

The development of nucleotides analogues, for the purpose of mimicking nucleic acids secondary structures, started with the pioneering work of Sekine with his approach toward *U*-turn loop, and then the design of conformationally constrained nucleotides grew up through the Nielsen's ring-closing metathesis pathway. Finally, the introduction of dioxaphosphorinane element at key positions along the sugar/phosphate backbone proved to be a rational concept to gain control on torsional angle sets. We have synthesized most of the possible members of the D-CNA family; all this structural units provide control on *α* to *ζ* torsional angle associated with a broad range of backbone conformations. Interestingly, the dioxaphosphorinane ring structures within D-CNAs are reasonably stable towards the oligonucleotide synthesis conditions according to a special care during the final deprotection step and are especially inert towards enzymatic degradation such as snake venon phosphodiesterase [69] as expected for phosphotriesters [70]. As a consequence, they are potential elements for the elaboration of synthetic nucleic acids with programmable folding and stability either in the double-helix or in the unpaired secondary structures. As a proof of concept, we demonstrated that relying on the restrain applied within oligodeoxynucleotides by means of *α*,*β*-D-CNA, high level of duplex formation ability or hairpin stabilization could be achieved. Therefore, at least with these leading components of the family, torsional stress applied to the sugar/phosphate backbone could be used in probing the necessary flexibility, or in contrary rigidity, of the nucleic acids architecture during the interaction with ligands or biomacromolecules. However, D-CNAs can be seen as a new alphabet for the conception of shape-defined nucleic acids, and if ten years ago C. Leumann [71] concluded a review by “*a large field that has not yet been tapped is the use of conformationally constrained nucleosides for the stabilization of secondary structural elements as, for example hairpin loops and bulges,*” there is still a long way before being able to properly address the use of each member of the family and to understand or predict the behavior of D-CNA within nucleic acids. Nevertheless, this new kind of nucleotide analogues could be the basis for the development of synthetic oligonucleotides for the modulation of protein/nucleic acids complex formation [72]. 

## Figures and Tables

**Table 1:** Summary of the backbone torsion angles derived from the canonical B_I_-, A-DNA duplex structures and of the synthesized D-CNA^a^.

Name	Isomer	Torsion angles					
		*α*	*β*	*γ*	*δ*	*ε*	*ζ*
B_I_-type		g^−^	t	g^+^	a^+^/t	t	g^−^/a^−^
A-type		g^−^	t	g^+^	g^+^/a^+^	a^−^/t	g^−^
*α*, *β*-D-CNA	(*R*_C5′_, *S*_*P*_)	g^−^	t				
	(*S*_C5′_, *R*_*P*_)	g^+^	t				
	(*R*_C5′_, *R*_*P*_)	a^+^	a^−^/t				
	(*S*_C5′_, *S*_*P*_)	a^−^	a^+^/t				
*α*, *β*-P-CNA	(*S*_C5′_, *S*_*P*_)	g^+^	t				
	(*R*_C5′_, *R*_*P*_)	a^−^/t	t				
LNA/*α*, *β*-D-CNA	(*R*_C5′_, *S*_*P*_)	g^−^	t		g^+^/a^+^		
	(*S*_C5′_, *R*_*P*_)	g^+^	t		g^+^/a^+^		
	(*R*_C5′_, *R*_*P*_)	a^+^	a^−^/t		g^+^/a^+^		
*α*, *β*, *γ*-D-CNA	*cis *	g^−^	g^−^	g^−^			
	*trans *	g^+^	c/g^+^	g^−^/a^−^			
*δ*, *ε*, *ζ*-D-CNA	(*S*_C4′_, *R*_*P*_)				a^+^/t	g^−^	g^−^
	(*S*_C4′_, *S*_*P*_)				a^+^/t	g^−^	a^+^/t
*v*_2_, *ε*, *ζ*-D-CNA	(*R*_C2′_, *S*_*P*_)				a^+^	g^+^	g^−^
	(*R*_C2′_, *R*_*P*_)				a^+^	g^+^	t
*ε*, *ζ*-D-CNA	(*S*_C3′_, *R*_*P*_)				c	t	g^+^
	(*S*_C3′_, *S*_*P*_)				c	t	g^−^

^a^The following 6-fold staggered pattern of the torsional angles is used: *cis*: 0 ± 30° (c), *gauche* (+) = 60 ± 30° (g^+^), *anticlinal* (+): 120 ± 30° (a^+^), *trans*: 180 ± 30° (t), *anticlinal* (−) = 240 ± 30° (a^−^), and *gauche* (−) = 300 ± 30° (g^−^). The notation a^+^/t is used to designate a torsion angle on the border of *anticlinal* (+) and *trans*.

**Table 2:** Sequences and melting temperatures of **CNAgm** containing duplexes.

Entry	Sequence^a^	*T*_*m*_^b^ (°C)	Δ*T*_*m*_ (°C)
**1**	5′-GCGTTTTTTGCT-3′ 3′-CGCAAAAAACGA-5′	49.0	—
**2**	5′-GCGTT***TxT ***TTGCT-3′ 3′-CGCAAAAAACGA-5′	55.0	+6.0
**3**	5′-GCGTT***TxTTxT ***GCT-3′ 3′-CGCAAAAAACGA-5′	59.0	+10.0
**4**	5′-GCG***TxTTxTTxT ***GCT-3′ 3′-CGCAAAAAACGA-5′	64.0	+15.0
**5**	5′-GCAAAAACTTGC-3′ 3′-CGTTTTTGAACG-5′	48.0	—
**6**	5′-GCAAAAAC***TxT ***GC-3′ 3′-CGTTTTTGAACG-5′	53.2	+5.2
**7**	5′-GCAAAAACTTGC-3′ 3′-CGTTT***TxT ***GAACG-5′	52.2	+4.2
**8**	5′-GCAAAAACTTGC-3′ 3′-CGTT***TxT ***TGAACG-5′	54.3	+6.3
**9**	5′-GCAAAAACTTGC-3′ 3′-CGT***TxT ***TTGAACG-5′	53.4	+5.4
**10**	5′-GCAAAAACTTGC-3′ 3′-CG***TxT ***TTTGAACG-5′	52.4	+4.4
**11**	5′-GCAAAAAC***TxT ***GC-3′ 3′-CGTTT***TxT ***GAACG-5′	55.9	+7.9
**12**	5′-GCAAAAAC***TxT ***GC-3′ 3′-CGTT***TxT ***TGAACG-5′	56.4	+8.4
**13**	5′-GCAAAAAC***TxT ***GC-3′ 3′-CGT***TxT ***TTGAACG-5′	57.6	+9.6
**14**	5′-GCAAAAAC***TxT ***GC-3′ 3′-CG***TxT ***TTTGAACG-5′	57.8	+9.8

^a^ ***TxT*** : (*R*_C5′_, *S*_P_) *α*, *β*-D-CNA TT (**CNAgm**) within the strand. ^b^UV melting experiments were carried out in sodium phosphate buffer (10 mM, pH 7.0) containing NaCl (100 mM) and EDTA (1 mM).

**Table 3:** Comparison of melting temperatures of **CNAgm** or **CNAgp** containing duplexes.

Entry	Sequence^a^	*T*_*m*_^b^ °C (Δ*T*_*m*_ °C)		
		*x* = PO_2_^−^	*x* = **C** **N** **A** **g** **m**	*x* = **C** **N** **A** **g** **p**
**1**	dA_14_/dT_4_ ***TxT ***T_4_	24.2	30.1 (+5.9)	10.5 (−13.7)
**2**	dA_14_/dT_6_ ***TxT ***T_6_	35.0	38.1 (+3.1)	24.0 (−11.0)
**3**	5′-GCGC***TxT ***GCCG-3′ 3′-CGCGAACGGC-5′	53.0	58.0 (+5.0)	44.0 (−9.0)
**4**	5′-ACAT***TxT ***GAAATGCAAATG-3′ 3′-TGTAAACTTTACGTTTAC-5′	49.0	52.0 (+3.0)	44.1 (−4.9)
**5**	5′-ACATTTGAAATGCAAATG-3′ 3′-TGTAAACTTTACG***TxT ***TAC-5′	49.0	54.9 (+5.9)	43.3 (−5.7)
**6**	5′-ACATTTGAAATGCAAATG-3′ 3′-TGTAAAC***TxT ***TACGTTTAC-5′	49.0	54.5 (+5.5)	49.0 (0.0)
**7**	5′-CTCATGAATATGCAAATC-3′ 3′-GAGTACTTATACG***TxT ***TAG-5′	52.4	55.0 (+2.6)	47.2 (−5.2)
**8**	5′-CTCATGAATATGCAAATC-3′ 3′-GAGTAC***TxT ***ATACGTTTAG-5′	52.4	56.5 (+4.1)	47.6 (−4.8)
**9**	5′-TGCTCAGTAAATAATGCA-3′ 3′-ACGAGTCATTTA***TxT ***ACGT-5′	55.6	59.0 (+3.4)	49.3 (−6.3)
**10**	5′-TGCTCAGTAAATAATGCA-3′ 3′-ACGAGTCA***TxT ***TATTACGT-5′	55.6	58.8 (+3.2)	49.6 (−6.0)
**11**	5′-ATCTCATTTGAAATGCAAATGGAA-3′ 3′-TAGAGTAAACTTTACG***TxT ***TACCTT-5′	57.9	59.3 (+1.4)	53.5 (−4.4)
**12**	5′-TGTCTCATGAATATGCAAATCACA-3′ 3′-ACAGAGTACTTATACG***TxT ***TAGTGT-5′	59.9	61.3 (+1.4)	55.7 (−4.2)
**13**	5′-CGCGAA***TxT ***CGCG-3′ 3′-GCGC***TxT ***AAGCGC-5′	62.0^c^	58.0 (−4.0)^c^	27.0 (−35.0) 82.0 (+20.0)

^a^ ***TxT*** : modified TT within the strand. ^b^UV melting experiments were carried out in sodium phosphate buffer (10 mM, pH 7.0) containing NaCl (100 mM) and EDTA (1 mM). ^c^Only one transition observed.

**Table 4:** Thermal melting temperatures (°C) of **CNAgp** within hairpin structures.

Entry	Sequence (5′-3′)^a^	*T*_*m*_ (°C)^b^	Δ*T*_*m*_ (°C)
**1**	ATCCTA *TTTT* TAGGAT	52.0	—
**2**	ATCCTA ***TxT *** *TT* TAGGAT	53.0	+1.0
**3**	ATCCTA *T* ***TxT *** *T* TAGGAT	55.0	+3.0
**4**	ATCCTA *TT * ***TxT *** TAGGAT	50.0	−2.0
**5**	ATCCTA *TTT* ***Tx *** **T** AGGAT	45.0	−7.0
**6**	AGGATCC *TTTT* GGATCCT	74.0	—
**7**	AGGATCC ***TxT *** *TT* GGATCCT	75.3	+1.3
**8**	AGGATCC *T* ***TxT *** *T* GGATCCT	77.5	+3.5
**9**	AGGATCC *TT* ***TxT *** GGATCCT	76.7	+2.7

^a^ ***TxT *** denotes an (*S*_C5′_, *R*_*P*_) *α*, *β*-D-CNA-modified TT dinucleotide (**CNAgp**) and italic character denotes loop nucleotide. Underlined bases indicate the loop-closing base pair. ^b^Melting temperatures (*T*_*m*_ values) were measured as the maximum of the first derivate of the UV melting curve (OD_260_ versus temperature, 20–90°C, 0.5°C/min) which was recorded at concentration around 5 μM in sodium phosphate buffer (10 mM, pH 7.0) containing NaCl (100 mM) and EDTA (1 mM).
